# Process-related predictors of readmissions and mortality following hip fracture surgery: a population-based analysis

**DOI:** 10.1007/s41999-020-00307-0

**Published:** 2020-03-26

**Authors:** Simo Sarimo, Hanna Pajulammi, Esa Jämsen

**Affiliations:** 1 Faculty of Medicine and Health Technology, Tampere University, FIN-33014 Tampere, Finland; 2 Central Finland Central Hospital, Central Finland Health Care District, Keskussairaalantie 19, FIN-40620 Jyvaskyla, Finland; 3 Tampere University Hospital, P.O.Box 2000, FIN-33521 Tampere, Finland

**Keywords:** Hip fracture, Orthogeriatrics, Readmission, Treatment protocol

## Abstract

**Purpose:**

Readmissions are common and complicate recovery after hip fracture. The objective of this study was to study readmission rates, factors associated with readmissions and effects of orthogeriatric liaison service in a setting where patients are discharged typically on the first postoperative day from the operating tertiary care hospital to lower-level health care units.

**Methods:**

A regionally representative cohort of 763 surgically treated hip fracture patients aged ≥ 50 years was included in this retrospective study, based on hospital discharge records. Primary outcome was a 30-day readmission, while the secondary outcome was a composite outcome, defined as readmission or death with a follow-up of 1 year at maximum.

**Results:**

The 30-day readmission rate was 8.3% and 1-year mortality was 22.1%. Short length of stay did not lead to poorer outcomes. Delay from admission to surgery of ≥ 4 days and discharge to primary health care wards were associated with an increased 30-day readmission rate. Age ≥ 90 years, delay to surgery, postoperative length of stay of ≥ 2 days and discharge on a Saturday were associated with higher risk for the composite outcome. Use of orthogeriatric liaison service at the operating hospital was associated with a lower risk of 30-day readmissions (11.8% vs. 6.2%, *P* = 0.012) whereas in longer follow-up readmissions seemed to cumulate similarly independent of orthogeriatric contribution. Patients living in the largest community in the area were discharged to a secondary care orthogeriatric ward and had a lower risk of 30-day readmissions than other patients (4.8% vs. 10.2%, *P* = 0.009).

**Conclusion:**

Use of orthogeriatric liaison service and later care at secondary care orthogeriatric ward seem to be beneficial for hip fracture patients in terms of reducing readmissions and mortality. Of the other care-related factors, short delay from admission to surgery and short total length of stay in the operating hospital was also associated with these outcomes, which, however, may relate to the effects of patient characteristics rather than the care process.

## Key summary points

**Aim:**

To study the readmission rates and predictors of readmission following hip fracture surgery in a tertiary care centre with the very short postoperative length of stay.

**Findings:**

Postoperative length of stay of only 1–2 days did not increase the risk of readmissions. Delay to surgery, prolonged length of stay, not receiving orthogeriatric contribution and discharge to primary rather than secondary care were associated higher readmission rate and/or mortality.

**Message:**

Although a very short stay in the operating hospital appears safe, hip fracture patients should not be discharged to primary care wards with insufficient resources for managing the acute postoperative phase.

## Introduction

Hip fractures are a common and costly injury worldwide [1]. In older patients, hip fractures have also been associated with increased morbidity [2] and increased health care costs [3], and increased mortality [4, 5]. From patients’ point of view, hip fracture often leads to decline in quality of life and functional ability and consequently there’s an increased need for assistance in daily living and many patients move to more assisted living environments [6, 7]. To avoid these adverse consequences, undelayed surgery, rapid mobilization and avoiding complications are essential [8–11]. Multidisciplinary orthogeriatric care improves the outcomes [12–15] but the arrangement of such services as well as access to such care vary between countries and regions.

A certain proportion of hip fracture patients are admitted back to hospital after the initial discharge from the operating unit. Such readmissions are harmful and, if possible, should be avoided, as they complicate and often prolong the recovery. Furthermore, in a US study the 1-year mortality for patients readmitted within 30 days was 56%, compared to 22% for those not readmitted [16].

In previous studies, the 30-day readmission rate has varied between 9 and 12% [16–20]. According to these studies, medical conditions cause readmissions more often than surgical reasons (90% vs. 7%, respectively) [20]**.** Pneumonia has been the most common single risk factor to cause readmissions in various studies [17, 18, 20]. Additionally, there is evidence that a pulmonary disease is a significant risk factor for future readmissions following a hip fracture [17, 20]. Also, a higher anesthesiologic risk score decreased functional ability, longer length of stay (LOS) and older age have been associated with a higher rate of readmissions [17, 18, 20, 21].

In Finland, hip fracture patients’ readmissions have been studied in a nationwide benchmarking project of the Finnish Institute for Health and Welfare. According to this register-based surveillance, among hip fracture patients operated in 2010–2016, 30-day readmissions occurred in 8.3–16.6% of cases and 30-day mortality was 3.6–7.9%, differing greatly between hospital districts [22]. These numbers suggest that the way how the treatment is arranged might influence hip fracture patients’ risk of readmission and death. A coordinated orthogeriatrics care model has previously been associated with a significant decrease in time to surgery, LOS, postoperative mortality rates and postoperative readmissions, and altogether every sixth readmission might be preventable by comprehensive treatment [23–25]. However, in previous studies the length of orthogeriatric intervention has ranged from days to weeks, whereas in our tertiary care center the LOS is only 1–2 days after which the patients are discharged to an orthogeriatric secondary care ward or to primary health care wards. There are some data concerning fast track hip fracture care with short postoperative hospitalization [26] but its effect on readmissions and value of geriatric contribution in the operating unit are unclear.

The objective of this study is to answer the following questions:

1. What percentage of patients are readmitted to the operating unit, how quickly their readmissions occurred, and what caused these readmissions?
2. Is short LOS (2 days or less) associated with an increased number of readmissions or deaths?
3. Does the risk of readmission or death vary according to the use of orthogeriatric liaison service?

## Methods

### Study population

This retrospective cohort study is based on electronic hospital discharge records gathered from Tampere University Hospital (TAUH), Tampere, Finland (population base ca. 530,000, 9% of which aged ≥ 75 years). The final study population comprised 763 patients aged ≥ 50 years who suffered a hip fracture (ICD-10 code S72.0, S72.1 or S72.2), were admitted between January 1, 2017 and June 30, 2018 and underwent hip fracture surgery (operation codes NFB10, NFB20, NFJ50, NFJ52 or NFJ54, according to Nordic Medico-Statistical Committee Classification of Surgical Procedures). Patients whose hip fracture was treated with total hip replacement were not included because they were operated in a separate hospital for joint replacement. The follow-up time ended on December 31, 2018 so every patient was observed for at least 6 months from discharge. The maximum follow-up was, however, set to 1 year.

**Fig. 1:**
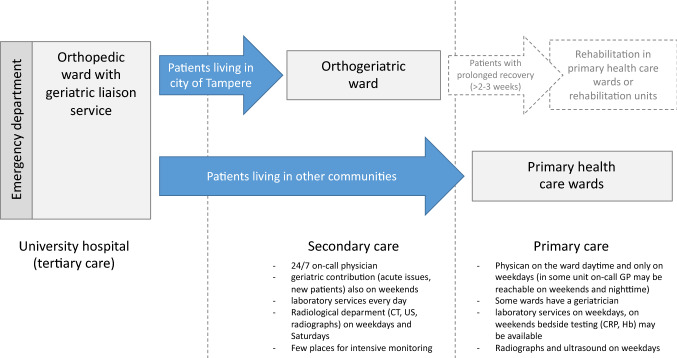
Management of hip fracture patients’ treatment in TAUH area

Orthogeriatric liaison service has been utilized in TAUH starting March 6, 2017, meaning that a geriatrician and a geriatric nurse participate in the treatment at orthopedic wards immediately after admission caused by a hip fracture. The geriatric team examines the patient’s background, screens for cognitive disorders, delirium, malnutrition, and frailty, performs clinical examination to identify comorbid acute conditions, and checks the medication list. Typical interventions include changes in medication (e.g., excess antihypertensive agents or diuretics, potentially inappropriate medications), prescription of energy-rich meals and protein supplements, advice and, if necessary, medications for the management of delirium. Geriatrician’s notes, including also recommendations for rehabilitation and possible memory testing or other examinations, are sent with the patient to the next treating unit (Fig. 1). This is of importance because only half of the communities in the district have geriatric services that contribute to care of hip fracture patients. Residents of the largest community in the area were discharged to a secondary care hospital with an orthogeriatric ward, 24/7 on-call physician and laboratory services on all weekdays. Residents of other communities were typically treated in primary health care wards lacking such services. Patients who were treated during the time periods when geriatric team was present (March 6, 2017 onwards, excluding vacation periods) were considered to have received orthogeriatric care by orthogeriatric liaison service.

### Data collection

The following data were extracted from mandatory electronic hospital discharge records: age, sex, municipality, date of admission, diagnoses, type of surgery, date of discharge, discharge destination and date of death. Data about readmissions were gathered by searching the electronic hospital discharge records for new hospital admissions, using patients’ unique social security numbers. A new admission was recognized as readmission, if the place of treatment was TAUH or the adjacent secondary care unit and the patient had been admitted within 365 days from discharge. Readmissions to the emergency department only (without following hospitalization) were excluded.

### Statistical analyses

The primary outcome in this study was a 30-day readmission, while the secondary outcome was a composite outcome, defined as a readmission or death with a follow-up time of 1 year at maximum. Incidence of readmissions was portrayed using Kaplan–Meier survival analysis. Factors predisposing for 30-day readmissions were analyzed using cross-tabulation. The differences were first tested using Pearson’s *χ*^2^ test, and then using logistic regression with adjustment for age, gender and type of fracture. Factors associated with the composite outcome were analyzed using Cox proportional hazards models both in univariate analysis and with the above-mentioned adjustments. The results of regression analyses are reported as odds ratios (OR) and hazard ratios (HR) with 95% confidence intervals. Analyses were conducted using IBM SPSS for Windows, version 23.0 (IBM Corp, Armonk, New York).

## Results

Data on 763 hip fracture patients were available (Table 1). Of the patients, 7 (0.9%) died during acute hospitalization and were excluded from further analyses.

**Table 1:** Study population

Patient and treatment characteristics	*n* (%) or median (range)
Age, years, median (range)	85 (51–104)
Age group	
< 75	134 (17.7)
75–79	97 (12.8)
80–84	132 (17.5)
85–89	242 (32.0)
90 or over	151 (20.0)
Gender	
Male	249 (32.9)
Female	507 (67.1)
Municipality	
Tampere	329 (43.5)
A municipality surrounding Tampere	177 (23.4)
Other municipality	250 (33.1)
Fracture type	
Femoral neck fracture	409 (54.1)
Pertrochanteric fracture	294 (38.9)
Subtrochanteric fracture	53 (7.0)
Type of surgery	
Hemiarthroplasty	389 (51.5)
Intramedullary nailing	321 (42.5)
Screw or plate fixation	46 (6.1)
Delay from admission to surgery	
0–1 days	519 (68.7)
2 days or more	237 (31.3)
Surgery on a weekday vs. weekend	
Surgery on a weekday	549 (72.6)
Surgery on weekend	207 (27.4)
Postoperative length of stay	
0–1 days	549 (72.6)
2 days or more	207 (27.4)
Total length of stay, days, median (range)	2 (0–12)
0–2 days	395 (52.2)
3 days or more	361 (47.8)
Orthogeriatric liaison service	
Used	538 (71.2)
Not used	218 (28.8)

The 30-day readmission rate was 8.3% and varied between 0% and 18.4% between months. 58 (7.6%) patients died within 1 month from discharge. 1-year mortality was 22.1% and varied between 16.3% and 39.0% between the observation months. During the first postoperative year, the composite outcome (readmission or death) occurred in 363 cases (48.0%). 26% of all readmissions (63/245) and 31% (51/167) of all deaths occurred during the first postoperative month. The median times to readmission and death were 97 (range 1–359) and 69 (range 1–362) days, respectively.

The most common reasons for readmission were pneumonia (*n* = 14, 5.7%), cardiac failure (*n* = 12, 4.9%) and acute tubulointerstitial nephritis (*n* = 8, 3.3%), followed by weakness and fatigue (*n* = 6, 2.4%), cerebral infarction caused by embolism (*n* = 6, 2.4%), atrial fibrillation or flutter (*n* = 6, 2.4%), intestinal obstruction (*n* = 5, 2.0%) and unspecified fever (*n* = 5, 2.0%). Altogether these 8 conditions accounted for 25.1% of all reasons for readmission.

### Factors predisposing for 30-day readmissions and combined outcomes

Factors associated with 30-day readmissions and 1-year composite outcomes are shown in Tables 2 and 3, respectively.

**Table 2:** The effects of patient and treatment characteristics on 30-day readmissions

Risk factor	30-day readmissions			Odds ratio (95% CI) adjusted for age, gender and fracture type	
	*n*/total	%	*P*	OR	95% CI
Gender					
Male	23/228	10.1	0.118	1	
Female	32/477	6.7		0.594	0.331–1.066
Age group					
< 75	11/129	8.5	0.767	1	
75–79	6/94	6.4		0.744	0.264–2.098
80–84	10/129	7.8		1.008	0.405–2.507
85–89	15/226	6.6		0.863	0.377–1.978
90 or over	13/127	10.2		1.457	0.609–3.485
Fracture type					
Femoral neck fracture	31/390	7.9	0.957	1	
Pertrochanteric fracture	20/268	7.5		0.979	0.543–1.765
Subtrochanteric fracture	4/47	8.5		1.129	0.379–3.366
Type of surgery					
Hemiarthroplasty	31/372	8.3	0.773	1	
Intramedullary nailing	20/288	6.9		0.264	0.037–1.899
Screw or plate fixation	4/45	8.9		0.428	0.058–3.145
Delay from admission to surgery*					
0–1 days	32/483	6.6	0.014	1	
2–3 days	20/212	9.4		1.458	0.812–2.617
4 days or more	3/10	30.0		6.408	1.554–26.423
Total length of stay					
0–2 days	23/370	6.2	0.099	1	
3 days or more	32/335	9.6		1.623	0.927–2.842
Postoperative length of stay					
0–1 days	37/516	7.2	0.302	1	
2 or more days	18/189	9.5		1.388	0.766–2.516
Day of discharge					
Monday	9/139	6.5	0.800	1	
Tuesday	8/129	6.2		0.936	0.349–2.513
Wednesday	8/98	8.2		1.244	0.461–3.358
Thursday	9/115	7.8		1.172	0.447–3.069
Friday	7/88	8.0		1.197	0.427–3.354
Saturday	6/74	8.1		1.234	0.420–3.628
Sunday	8/62	12.9		2.026	0.738–5.564
Discharge on a weekday vs. weekend					
Discharge on a weekday	41/569	7.2	0.228	1	
Discharge on weekend	14/136	10.3		1.453	0.765–2.759
Day of surgery					
Monday	13/114	11.4	0.558	1	
Tuesday	8/95	8.4		0.719	0.284–1.820
Wednesday	8/114	7.0		0.557	0.220–1.408
Thursday	6/86	7.0		0.561	0.203–1.551
Friday	8/105	7.6		0.618	0.224–1.565
Saturday	8/86	9.3		0.801	0.315–2.037
Sunday	4/105	3.8		0.303	0.095–0.965
Surgery on a weekday vs. weekend					
Surgery on a weekday	43/514	8.4	0.359	1	
Surgery on weekend	12/191	6.3		0.748	0.385–1.454
Municipality*					
Tampere	15/311	4.8	0.009	1	
Other municipality	40/394	10.2		2.186	1.179–4.055
Orthogeriatric liaison service*					
Used	31/501	6.2	0.012	1	
Not used	24/204	11.8		2.030	1.156–3.566

Patients who died within 30 days from discharge (51 cases) were excluded from 30-day readmission assessment

*p < 0.05

**Table 3:** The effects patient and treatment characteristics on the composite outcome (readmission or death in 1-year follow-up)

Risk factor	Composite outcome				
	*n* (%)	Unadjusted		Adjusted for age, gender and fracture type	
		HR	95% CI	HR	95% CI
Gender					
Male	123 (49.4)	1		1	
Female*	240 (47.3)	0.889	0.716–1.105	0.784	0.626–0.982
Age group					
< 75	58 (43.3)	1		1	
75–79	42 (43.3)	1.039	0.698–1.545	1.068	0.717–1.590
80–84	60 (45.5)	1.091	0.761–1.566	1.180	0.818–1.704
85–89	109 (45.0)	1.098	0.798–1.510	1.180	0.853–1.633
90 or over*	94 (62.3)	1.786	1.287–2.478	1.920	1.371–2.689
Fracture type					
Femoral neck fracture	186 (45.5)	1		1	
Pertrochanteric fracture	148 (50.3)	1.167	0.941–1.449	1.202	0.968–1.493
Subtrochanteric fracture	29 (54.7)	1.312	0.887–1.941	1.302	0.879–1.928
Type of surgery					
Hemiarthroplasty	175 (45.0)	1		1	
Intramedullary nailing	164 (51.1)	1.213	0.980–1.501	1.320	0.713–2.444
Screw or plate fixation	24 (52.2)	1.157	0.755–1.773	1.374	0.753–2.506
Delay from admission to surgery					
0–1 days	250 (48.2)	1		1	
2–3 days	104 (46.2)	1.003	0.798–1.261	1.002	0.797–1.259
4 days or more*	9 (75.0)	1.997	1.027–3.885	1.997	1.026–3.888
Total length of stay					
0–2 days	176 (44.6)	1		1	
3 days or more*	187 (51.8)	1.237	1.007–1.520	1.231	1.002–1.513
Postoperative length of stay					
0–1 days	245 (44.6)	1		1	
2 or more days*	118 (57.0)	1.369	1.099–1.705	1.343	1.076–1.675
Day of discharge					
Monday	61 (41.5)	1		1	
Tuesday	64 (46.7)	1.185	0.835–1.683	1.152	0.811–1.637
Wednesday	52 (47.7)	1.279	0.883–1.852	1.288	0.889–1.866
Thursday	60 (49.2)	1.311	0.918–1.872	1.260	0.881–1.801
Friday	46 (47.9)	1.246	0.850–1.827	1.193	0.813–1.750
Saturday*	36 (52.9)	1.552	1.053–2.287	1.511	1.024–2.229
Sunday	36 (48.0)	1.501	0.994–2.267	1.417	0.937–2.143
Discharge on a weekday vs. weekend					
Discharge on a weekday	283 (46.3)	1		1	
Discharge on weekend	80 (55.2)	1.287	1.004–1.650	1.260	0.982–1.616
Day of surgery					
Monday	58 (48.3)	1		1	
Tuesday	55 (51.9)	1.131	0.782–1.635	1.142	0.789–1.651
Wednesday	60 (49.2)	1.023	0.713–1.468	0.996	0.693–1.430
Thursday	44 (47.8)	1.014	0.685–1.500	0.999	0.674–1.479
Friday	59 (54.1)	1.118	0.778–1.606	1.098	0.763–1.579
Saturday	46 (48.9)	1.001	0.680–1.474	1.005	0.682–1.479
Sunday	41 (36.3)	0.691	0.463–1.030	0.682	0.457–1.020
Surgery on a weekday vs. weekend					
Surgery on a weekday	276 (50.3)	1.017	0.827–1.250	1.270	0.998–1.617
Surgery on weekend	87 (42.0)	1		1	
Municipality					
Tampere	165 (50.2)	1		1	
Other	198 (46.4)	0.983	0.800–1.209	0.962	0.781–1.186
Orthogeriatric liaison service					
Used	253 (47.0)	1		1	
Not used	110 (50.5)	1.130	0.904–1.414	1.137	0.909–1.423

HR with 95% CI using unadjusted variables and variables adjusted for age, gender and fracture type. Composite outcomes were investigated using Cox hazards model

*p < 0.05

**Fig. 2:**
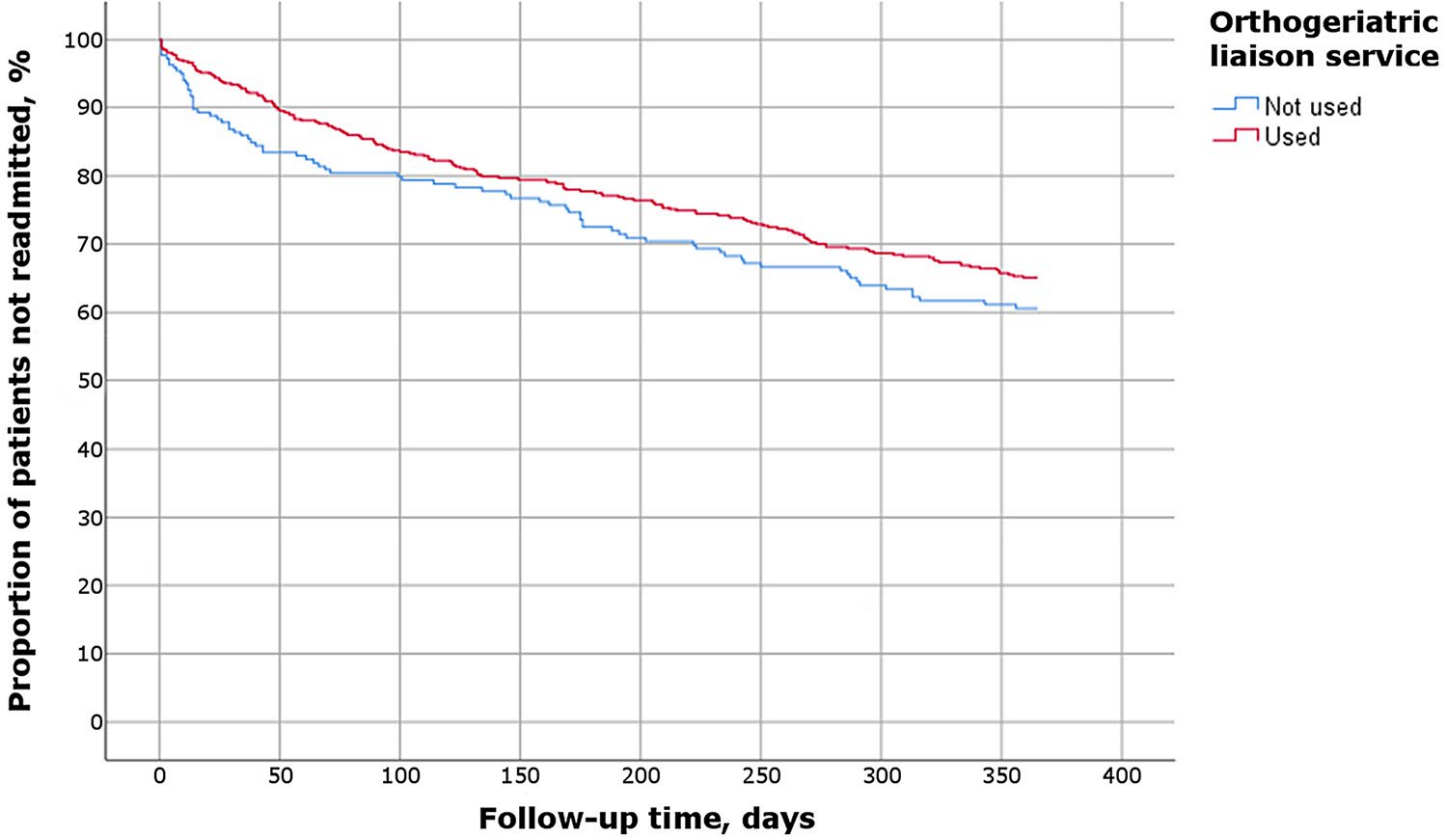
Kaplan–Meier survival analysis regarding readmissions in 365-day follow-up, according to the use of orthogeriatric liaison service

30-day readmissions were more frequent among patients who underwent surgery on the 4th day or later (30.0% vs. 9.4% in patients operated earlier, *P* = 0.014; adjusted OR 6.408, 95% CI 1.554–26.423), who lived in other municipalities than the city of Tampere (10.2% vs. 4.8%, *P* = 0.009; adjusted OR 2.186, 95% CI 1.179–4.055) and who did not receive orthogeriatric liaison service (11.8% vs. 6.2%, *P* = 0.012; adjusted OR 2.030, 95% CI 1.156–3.566) (Table 2). The effect of orthogeriatric liaison service was the most beneficial during the early follow-up whereas in longer follow-up readmissions seemed to cumulate similarly independent of orthogeriatric contribution (Fig. 2).

Composite outcome (readmission or death) was more common among patients aged ≥ 90 years (adjusted HR 1.920, 95% CI 1.371–2.689, compared to patients aged < 75 years), patients with a delay to surgery of ≥ 4 days (adjusted HR 1.997, 95% CI 1.026–3.888, compared to delay of 0–1 days), patients with a LOS of 3 days or more (adjusted HR 1.231, 95% CI 1.002–1.513), patients discharged on the second postoperative day or later (adjusted HR 1.343, 95% CI 1.076–1.675) and patients discharged on a Saturday (adjusted HR 1.511, 95% CI 1.024–2.229).

## Discussion

In this regionally representative analysis, a long delay from admission to surgery was associated with an increased 30-day readmission rate whereas the use of orthogeriatric liaison service in the operating university hospital was associated with a decreased risk. A long delay to surgery was also linked with a higher frequency of the composite outcome (readmission or death in 1-year follow-up). Although the patients were discharged from the operating hospital very early (73% by the end of the first postoperative day), such short LOS was not related to an increased number of 30-day readmissions. Instead, especially considering the combined outcome, the result was the opposite: prolonged LOS was linked to more readmissions or deaths in a year’s time span. Patients discharged on Saturdays had a significant increase in the rate of the composite outcome whereas such increase was not evident in 30-day readmissions. Although patients receiving orthogeriatric liaison service had less 30-day readmissions than others, there was no difference in the 1-year composite outcome.

The 30-day readmission rate in our study was 8.3%, which represents the lowest rates reported in Finland (years 2010–2016) although we included also second hip fractures and patients living in nursing homes and institutions who are excluded from the nationwide surveillance [22]. Despite the short stay in the operating hospital, our 30-day readmission rate was slightly lower than in previous studies [16–20]. As it comes to 1-year mortality, the 22.1% mortality is well within the wide range (8.4–36%) reported in previous studies [27] and is in line with recent earlier observations from another part of Finland [6]. Contradicting some earlier studies [18, 27–29], gender had no effect on the occurrence of readmissions and death, and age did not affect 30-day readmission rate.

Supporting earlier observations [8, 9, 11, 30], a short delay (0–1 days) from admission to surgery had a connection to both lower 30-day readmission rates and fewer 1-year outcomes. A recent study suggests that a delay in hip fracture surgery for more than 12 h after admission impairs 30-day survival especially among patients with severe systemic disease [8] whereas the opposite has been reported as well [30]. As reasons for the delay were not recorded, these observations could not be confirmed. Furthermore, in the lack of clinical details, it cannot be precluded that the association between short delay and the outcomes is at least partly related to patient characteristics (e.g., fitness for surgery) rather than the care process as such. Use of direct oral anticoagulants and platelet inhibitors represent an increasingly common reason for delaying surgery [31] but in our materials, such drugs were used by a relatively small number of patients (approximately 5%, unreported observation) and they do not seem to increase 30-day mortality [31].

An important and new observation in the present study was that the very short LOS of 2 days or less was not associated with more frequent undesirable outcomes although a large share of patients was discharged to primary care wards with often limited possibilities for intensive treatments and lacking on-call physician and physician service on weekends. This result is in line with a Danish study where 30-day readmission rates were similar (ca. 12%) in fast-track group and conventional care group (LOS approximately 5 days in both groups) [26]. On the other hand, it is possible that patients who had short LOS were healthier and hence better suited for surgery and discharge without additional interventions, which could explain both LOS as well as lower 30-day readmission rate. Interestingly, if a patient was discharged on a Saturday, there was a higher risk for the composite outcome. There are two possible explanations for this association. First, it might be because orthogeriatric liaison service was not available on weekends, and the patient was not necessarily met on Friday due to the surgery. Secondly, patients discharged to primary care units most likely are not evaluated by a physician until next Monday which might cause delays in the care and management of possible acute conditions. This explanation is supported by the observation that patients living in the largest community and discharged to a secondary care hospital with 24/7 services had significantly lower rates of 30-day readmissions and composite outcome. On the other hand, it must be acknowledged that the association between postoperative LOS and increased mortality [32] may be for example due to complications and other health issues, which might also affect our findings considering LOS.

Patients who did not receive orthogeriatric liaison service had more 30-day readmissions, but the same result was not evident using 1-year composite outcome. This could be interpreted as orthogeriatric treatment especially preventing early readmissions while having a lesser impact with longer follow-up. According to one previous study, orthogeriatric treatment has been associated with decreased mortality and a lower readmission rate [33] whereas such association has not been reported in other studies [2], including a randomized controlled trial [14]. In all these studies, the orthogeriatric intervention has lasted longer than in our study (i.e., for 1.5–2 weeks), and we are not aware of earlier studies concerning the effect of very short orthogeriatric intervention, like ours, on readmission rates, mortality or clinical outcomes. In terms of mortality, orthogeriatric care at an orthogeriatric ward, instead of shared care of consultations at orthopedic ward, seems to be more effective [25, 34]. In our study, a large share of patients was treated in such ward in a secondary care hospital, and these patients had a lower readmission rate than others whereas there was no difference in the 1-year outcomes. Therefore, it is probable that the contribution of the orthogeriatric ward was beneficial for these patients. However, a greater study population is needed to further analyze the impact of the orthogeriatric ward at secondary care as well as the combined effect of liaison service and later care at geriatrician-led ward. Altogether, our results suggest that hip fracture patients’ care in a unit able to meet all the patients’ needs is beneficial in preventing early readmissions. In longer follow-up, new hospital admissions are frequent which is understandable considering the high age and multimorbidity in this patient group, and so it feels unlikely that orthogeriatric care would dramatically affect the outcomes in longer follow-up.

The strengths of this study include a regionally representative cohort of consecutive hip fracture patients. Patients treated using total hip replacement were not included in this study due to regional treatment protocols. Furthermore, coding of diagnoses in the discharge records may sometimes reflect geriatric patients’ comorbidities and the reason for readmission is not always coded in detail. As patient records (medical charts) were not reviewed in this study, the exact reasons and factors contributing to the outcomes unfortunately remain unclear. It is possible that differences in patients’ morbidity between different communities might explain the difference between the largest community and others. On the other hand, it is possible that some patients may not have been readmitted due to long distance (> 1.5 h ambulance ride) from the distant communities, which would lead to underestimation of readmission rates. It is, however, unlikely that such selection would have affected the results concerning orthogeriatric liaison service, delay to surgery or LOS. Due to insufficient patient numbers, community-level analyses about the value of orthogeriatric liaison service could not be performed, however.

## Conclusion

Care-related factors such as the use of orthogeriatric liaison service and arrangement of later care at secondary care orthogeriatric ward, short delay from admission to surgery, and short total length of stay, that can be beneficial to hip fracture patients aged 50 years or older in terms of reducing readmissions and mortality. Although in general very short stay in tertiary care hospital appears safe, hip fracture patients should not be discharged to primary care wards with insufficient resources for managing hip fracture patients in the acute postoperative phase. Further study is required to specify how hip fracture patients’ treatment should be organized and if readmissions could be reduced using specific discharge criteria.
